# From pulse width modulated TENS to cortical modulation: based on EEG functional connectivity analysis

**DOI:** 10.3389/fnins.2023.1239068

**Published:** 2023-08-02

**Authors:** Armita Faghani Jadidi, Winnie Jensen, Ali Asghar Zarei, Eugen Romulus Lontis, S. Farokh Atashzar

**Affiliations:** ^1^Center for Neuroplasticity and Pain (CNAP), Department of Health Science and Technology, Aalborg University, Aalborg East, Denmark; ^2^Department of Electrical and Computer Engineering, New York University, New York, NY, United States; ^3^Department of Mechanical and Aerospace Engineering, New York University, New York, NY, United States; ^4^Department of Biomedical Engineering, New York University, New York, NY, United States; ^5^NYU WIRELESS, New York University (NYU), New York, NY, United States; ^6^NYU Center for Urban Science and Progress (CUSP), New York University (NYU), New York, NY, United States

**Keywords:** modulated TENS, functional brain connectivity, graph theory, pain, neurorehabilitation

## Abstract

Modulation in the temporal pattern of transcutaneous electrical nerve stimulation (TENS), such as Pulse width modulated (PWM), has been considered a new dimension in pain and neurorehabilitation therapy. Recently, the potentials of PWM TENS have been studied on sensory profiles and corticospinal activity. However, the underlying mechanism of PWM TENS on cortical network which might lead to pain alleviation is not yet investigated. Therefore, we recorded cortical activity using electroencephalography (EEG) from 12 healthy subjects and assessed the alternation of the functional connectivity at the cortex level up to an hour following the PWM TENS and compared that with the effect of conventional TENS. The connectivity between eight brain regions involved in sensory and pain processing was calculated based on phase lag index and spearman correlation. The alteration in segregation and integration of information in the network were investigated using graph theory. The proposed analysis discovered several statistically significant network changes between PWM TENS and conventional TENS, such as increased local strength and efficiency of the network in high gamma-band in primary and secondary somatosensory sources one hour following stimulation. Our findings regarding the long-lasting desired effects of PWM TENS support its potential as a therapeutic intervention in clinical research.

## 1. Introduction

Transcutaneous electrical nerve stimulation has been widely suggested as a non-invasive and drug-free therapeutic intervention for several physiological conditions, including pain alleviation depending on stimulation characteristics (Khadilkar et al., 2008; Chen and Johnson, 2009; Tan et al., 2016; Peng et al., 2019). For instance, it recently has been shown that conventional high-frequency (HF) TENS with low intensity leads to higher analgesic effects for acute experimental pain than “acupuncture-like” TENS with low frequency and high intensity (Peng et al., 2019). Furthermore, literature has revealed the positive contribution of conventional TENS in pain alleviation for patients with chronic pain, e.g., back pain (Khadilkar et al., 2008), and phantom limb pain (PLP) (Katz and Melzack, 1991; Mulvey et al., 2013; Hu et al., 2014; Johnson et al., 2015). While clinical studies validated the effectiveness of TENS intervention in chronic pain and amputees with PLP, the underlying mechanism of TENS is still under investigation. Researchers believe that the TENS-induced analgesic effect is correlated with gate control theory at the spinal level (Melzack and Wall, 1965), facilitation of the corticospinal (CS) pathway (Gunduz et al., 2020; Faghani Jadidi et al., 2022), or cortical inhibition at the somatosensory cortex (Ploner et al., 2017; Peng et al., 2019; Zarei et al., 2019, 2021).

Several brain areas are mainly involved in pain and sensory translation and regulation, namely, the primary and secondary somatosensory cortex (SI and SII), the medial prefrontal cortex (PFC), the anterior cingulate cortex (ACC), and the insular cortex (Apkarian et al., 2005; Stern et al., 2006; Kregel et al., 2015; Makin and Flor, 2020; Zheng et al., 2020). Neuronal oscillation in pain-related brain areas at different frequency bands is believed to play a major role in chronic pain onset (Ploner et al., 2017; Ebrahimian et al., 2018; Arendsen et al., 2021). Human studies in line with animal-based research have revealed the significant increase in theta, alpha, beta, and gamma oscillations in chronic pain, mostly in the medial prefrontal cortex and somatosensory cortices (Sarnthein et al., 2006; Stern et al., 2006; Leblanc et al., 2014, 2016; Ploner et al., 2017). In this regards, previous studies have shown that TENS results in a suppression of cortical oscillations from theta to low beta frequency bands strengthening TENS mechanism in pain alleviation (Ebrahimian et al., 2018; Peng et al., 2019; Zarei et al., 2021).

Functional cortical plasticity and network reorganization are also believed to actively contribute to chronic pain. Investigations based on fMRI and EEG data have shown alterations in both inter-hemispheric and intra-hemisphere functional connectivity (FC) within and beyond the somatosensory cortex in chronic pain patients (Ta Dinh et al., 2019; Makin and Flor, 2020; Zheng et al., 2020; Kandić et al., 2021). Several indices have been used to quantify the functional connection between brain regions both in frequency domain [such as phase lack value (PLV) (Aydore et al., 2013; Mheich et al., 2015)] and time domain [such as Spearman correlation (Pizzagalli et al., 2003; Savva et al., 2019)]. Furthermore, graph theory has also been suggested as an approach for investigating topological properties of the brain FC network regarding information flow between brain regions in patients with the neurological disease (Zhang et al., 2015; Damborská et al., 2020) or chronic pain (Zheng et al., 2020; Lenoir et al., 2021; Zarei et al., 2022). Interestingly, recent studies have shown a significant effect of conventional TENS intervention on functional brain connectivity at both regions and network level, supporting the TENS’s effectiveness in acute and chronic pain relief (Peng et al., 2019; Zarei et al., 2022).

Motivated by the observed benefits, there are currently active researches in the literature to find more effective alternative TENS patterns (Grill, 2018), such as burst (Schu et al., 2014; Tilak et al., 2016) and pulse width modulated (PWM) (Tan et al., 2016; Bouafif and Ellouze, 2018). PWM TENS has been tested on low back patients, and the results revealed a similar analgesic effect to conventional TENS induced effect, while patients reported that the therapy with PWM TENS was more pleasant (Tan et al., 2016). Dynamic recruitment of fibers due to varying pulse width leads to neural activation more similar to physiological signals resembling natural sensation, which is mentioned as a possible explanation for the more pleasant sensation experience (Tan et al., 2014). In addition, recent article studied the effects of PWM TENS on the CS pathway (Faghani Jadidi et al., 2022). The results revealed greater facilitation in the CS pathway and corticomotor map compared with conventional TENS. These alterations are considered as desired effects on the central nervous system that may lead to chronic pain alleviation. Moreover, suppression of sensory evoked potentials (SEPs) at S1 following PWM TENS was accompanied by a significant reduction of perceived sensation intensity (Jadidi et al., 2023). However, the PWM TENS mechanism on the cortical network level needs further investigation. Therefore, we conducted a novel study to compare the possible induced changes in the cortical network consisting of pain-related cortical regions, following modulated (PWM) and non-modulated (HF) TENS in healthy subjects. The study aimed to investigate possible cortical network biomarkers by PWM TENS which can support the potential of this pattern as alternative stimulation in pain therapy.

## 2. Materials and methods

**Figure 1:**
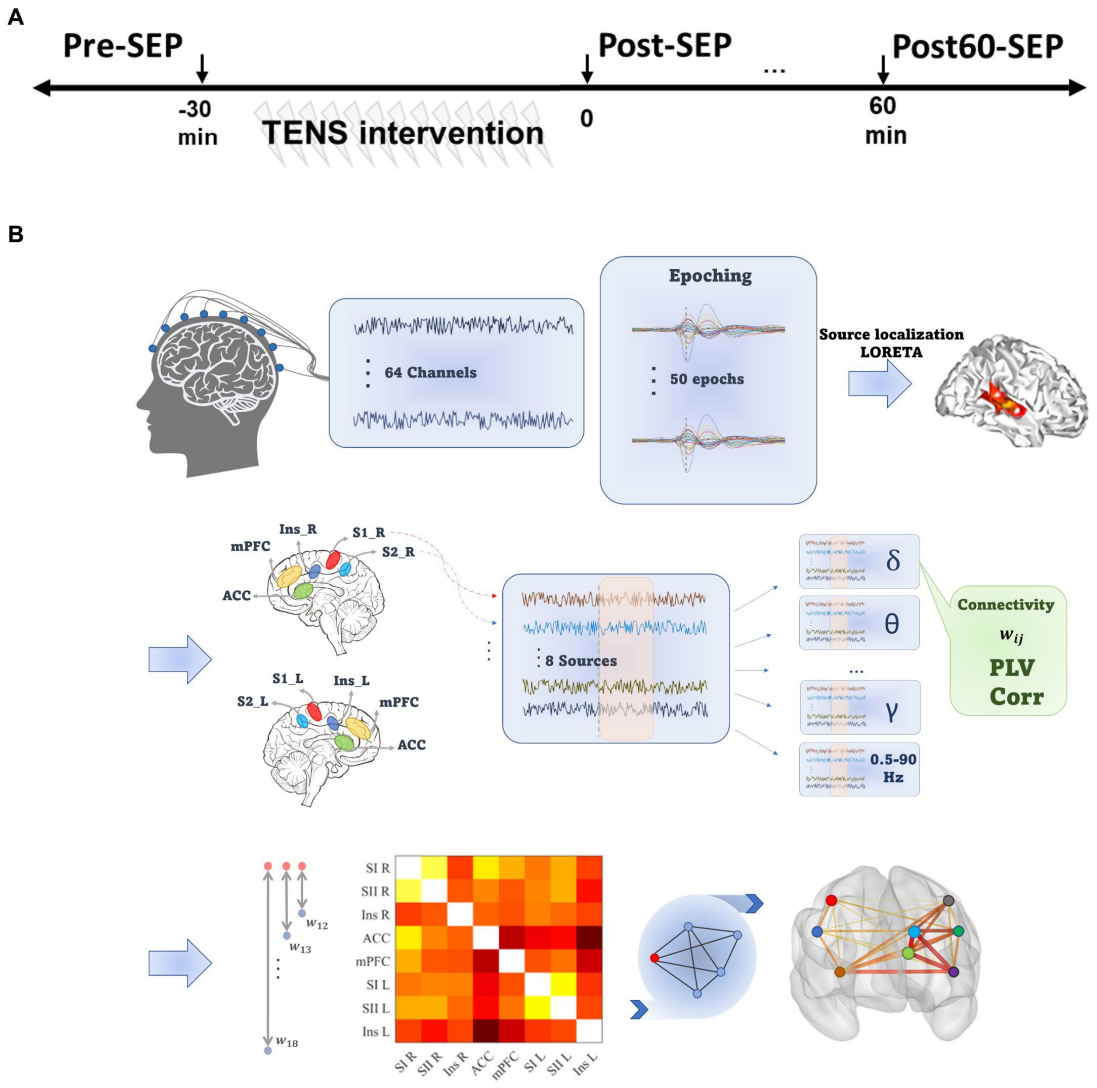
Experimental and data processing outline. **(A)** The experimental procedure consisted of three EEG recording phases at baseline (Pre), immediately and 60 min after TENS (Post and Post60, respectively). **(B)** Data processing steps. For each Subject, pre-processed EEG signal was divided into 2 s epochs, and LORETA was implemented to localize the sources of activity. Eight pain-related regions were selected, and functional connectivity between all pairs of selected ROIs was quantified based on phase and amplitude synchronization in seven frequency bands. Eventually, the cortical network consisted of ROIs as nodes and the mean of PLV or Cor values across epochs defined edges.

An overview of the experimental procedures is shown in Figure 1A. Each experimental session consisted of four phases, including the intervention phase where the TENS pattern was delivered and three recording phases before (Pre), immediately (Post), and 60 min after (Post60) the intervention phase.

### 2.1. Participants

Twelve healthy right-handed subjects (seven women, age = 26.6 ± 2.7 years) participated in this study. They all completed two separate experimental sessions, each lasting 3.5 h. Subjects had no history of peripheral or central nervous system disease or contraindications to tolerating electrical stimulation (e.g., having pacemakers). All participants signed the informed consent form and the experimental protocol was approved by The North Denmark Region Committee on Health Research Ethics (N-20190016) in accordance with the Declaration of Helsinki.

### 2.2. TENS intervention

Each subject received both stimulation patterns in a random order consisting of bipolar rectangular pulses with the characteristics as follows; (1) HF: Conventional high-frequency TENS (100 Hz) with a constant pulse width of 500 μs as control group and (2) PWM: High-frequency TENS (100 Hz) with a dynamic pulse width (modulating sinusoidal wave of 1 Hz) that varied between 0 to 500 μs (Faghani Jadidi et al., 2022; Jadidi et al., 2023). Each pattern was delivered for 30 min (20 s on, 10 s off (Mang et al., 2010; Ishibashi et al., 2021) with an intensity set individually at 80% of the discomfort threshold (with no visual movement) (Zarei et al., 2021, 2022; Faghani Jadidi et al., 2022). The electrical charges from a current stimulator (DS5, Digitimer, UK) were applied to the skin through a pair of oval electrodes (contact size of 4 × 6, Dura Stick premium) positioned over the median nerve of the dominant hand.

### 2.3. Data collection

Over the recording phase, subjects were comfortably seated in an armchair and asked to keep their gaze at a plus sign located on the screen in front of them. The continuous EEG was collected from 64 channels (actiCAP, Brain Products GmbH, Germany) positioned according to the international 10–20 system and connected to a BrainAmp MR plus amplifier (Brain Products, GmbH). The pre-amplified EEG signal was sampled at 5 kHz. The impedance of electrodes was initially monitored and was maintained below 25 kΩ. FCz was selected as the reference channel.

To assess the effect of TENS on the cortical signals, series of single square-wave pulses with 500 μs pulse width were delivered to the median nerve of the right hand to elicit sensory evoked potentials (SEP) signals. It was applied as two sets of 25 pulses with 15 s interstimulus intervals at each time phase (Pre, Post, and Post60) to avoid habituation and fatigue. The intensity of pulses was adjusted at 2.5 times the individual sensory threshold measured at the beginning of the experiment day using the staircase procedure (Faghani Jadidi et al., 2022).

### 2.4. Data pre-processing

The preprocessing steps (illustrated in Figure 1B) were implemented in the BrainVision Analyzer software (Version 2.2.2 Brain Products, GmbH). The continuous EEG was downsampled (256 Hz) and re-referenced to the averaged reference. Next, filtered signals between 0.5 to 90 Hz (IIR bandpass filter, 4th order Butterworth) were divided into 2 s epochs (from 500 ms prior to 1,500 ms after stimulus). Next, blink-contaminated epochs were detected using independent component analysis (ICA) and discarded from further analysis. The spatial resolution of the remaining epochs was enhanced using the Laplacian current source density (CSD), which reduces the negative impact of volume conduction and saccadic eye movement effects on phase synchronization (Kayser and Tenke, 2015). Filtered epochs were baseline corrected by subtracting an average of the activity in a 500 ms interval preceding the stimulus. Data with amplitude exceeding ± 100 mv were excluded as epochs contaminant by non-physiological noise.

### 2.5. Source localization

To isolate and localize the cortical areas as nodes in the functional brain connectivity network, the low-Resolution Electromagnetic Tomography (LORETA) algorithm was applied in this paper on each epoch (Brainvision analyzer 2.2). The LORETA computes and estimates the source activities and reduces the dimensionality of the data from the 64-channel electrode space to few cortical regions’ activations (Pascual-Marqui et al., 1994). Several studies have previously validated the consistency of the LORETA algorithm in physiological conditions (Stern et al., 2006; Jiang et al., 2019; Zarei et al., 2022). The head model was set based on the Montreal Neurological Institute template (MNI-305), including 2,394 voxels with 7 cubic mm spatial resolution. Cortical regions of interest (ROIs) were specified based on the Automated Anatomical Labeling (AAL) brain template (Collins et al., 1998).

Accordingly, to generate the FC network, activities from eight pain-related ROIs, consisting of primary and secondary sensory cortex in the left (SI_L and SII_L) and the right hemispheres (SI_R and SII_R), mPFC, ACC, and Insula in both hemispheres, were estimated. The selected regions are based on the literature on pain processing (Figure 1B). The source activity in these ROI’s were calculated by averaging the current density value of all voxels within the ROI, and computed for eight predefined frequency bands [delta: 0.5–4 Hz, theta: 4–8 Hz, alpha: 8–13 Hz, beta: 14–40 Hz, gamma: 40–49 Hz, high gamma: 51–90 Hz and 0.5–90 Hz (Tottrup et al., 2020; Zarei et al., 2022)].

### 2.6. Functional connectivity

In neuroscience, various methods have been used to estimate the functional connectivity between various brain regions. The overall understanding is that when two areas of the brain are functionally coupled, the activations may represent some format of synchronization which can be quantified to evaluate the connectivity strength.

Phase lag value (PLV): Among frequency-domain methods, PLV is a common metric for assessment of the degree of phase synchronization between estimated instantaneous phases of two neural traces (Aydore et al., 2013; Schmidt et al., 2014; Mheich et al., 2015). For this, the Hilbert transform was implemented on each epoch, and the PLV between the neural activity of two brain sources was calculated as follows:

[1] $$PLV=|e^{j\Delta \varphi \left(t_{p}\right)}|$$

Where Δφ denotes the phase difference between two sources activity at t_p_ point.

Spearman correlation: In addition to frequency domain techniques, time-domain methods have also been used for the assessment of FC. In this regard, temporal Spearman’s correlation between the neural activity of two sources is a common nonparametric method to measure FC between pair of brain regions (Pizzagalli et al., 2003; Savva et al., 2019). Compared to Pearson’s correlation, Spearman’s correlation is more reliable for estimating nonlinear and nonparametric coupling between the two signals (Tabatabai et al., 2021).

In this work, based on PLV and Spearman’s correlation, we established two FC matrixes named PLV_FC_ and Cor_FC_, respectively, by averaging metrics across all epochs for each frequency band, time phase, and participant.

### 2.7. Graph analysis

Comparative analysis between induced alteration in brain network following two TENS patterns was performed in this study using graph analysis. The networks in the present work consisted of nodes (i.e., selected brain regions) and pairwise functional connectivity using PLV and Spearman’s correlation as edges. To represent the segregation and integration of the brain FC network, three graph metrics was extracted, namely node strength, efficiency (EF), and clustering coefficient (CC). The strength of the node is defined as the sum of the edges connecting to the desired node, and thus, global network strength represents the overall strength of network connectivity (Yuan et al., 2013). Global network efficiency depicts functional integration by estimating the quality of information transmission in a network. It is considered as the average of inverse shortest path lengths between all pairs of nodes. The local efficiency is similarly calculated by inversing the average shortest path between selected node and the rest of the neighbor nodes (Bullmore and Sporns, 2009). Furthermore, the cluster coefficient calculates the fraction of triangular connected nodes neighboring the node of interest. Global cluster coefficient is defined as the average of local values within a network (Bullmore and Sporns, 2009).

The local network metrics were extracted from phase-based and correlation-based network named PLV_L_G_ and Cor_L_G_, respectively, for each ROI, frequency band, and participants at Pre, Post, and Post60 levels. Eventually, the global node strength, efficiency, and clustering coefficient were also calculated from PLV_FC_ and Cor_FC_ (termed Cor_G_G_ and PLV_G_G_, respectively) for each TENS pattern, frequency band, and time phase.

### 2.8. Statistical analysis

The statistical analysis was conducted in RStudio (R version 4.0.3). To compare the effect of two TENS patterns on topology characteristics of the cortical network, the difference of post phases measurements (Post and Post60) and Pre values were calculated for each extracted feature (PLV_L_G_, Cor_L_G_, PLVG_G, and Cor_G_G_), frequency band, and TENS patterns. Next, the normality of the network’s values in each new time phase (Post - Pre ad Post60 - Pre) was examined by the Shapiro test. Due to the non-normal distribution of data, the Wilcoxon test was implemented on each subtracted global metrics in new time phases and frequency bands as main dependent variables and “pattern” (PWM and HF) as a between-subject factor. The same procedure was conducted for local metrics with brain regions as an extra condition for comparative analysis. *p*-values were Bonferroni-corrected and the significance level was remained as *p* < 0.05.

## 3. Results

**Figure 2:**
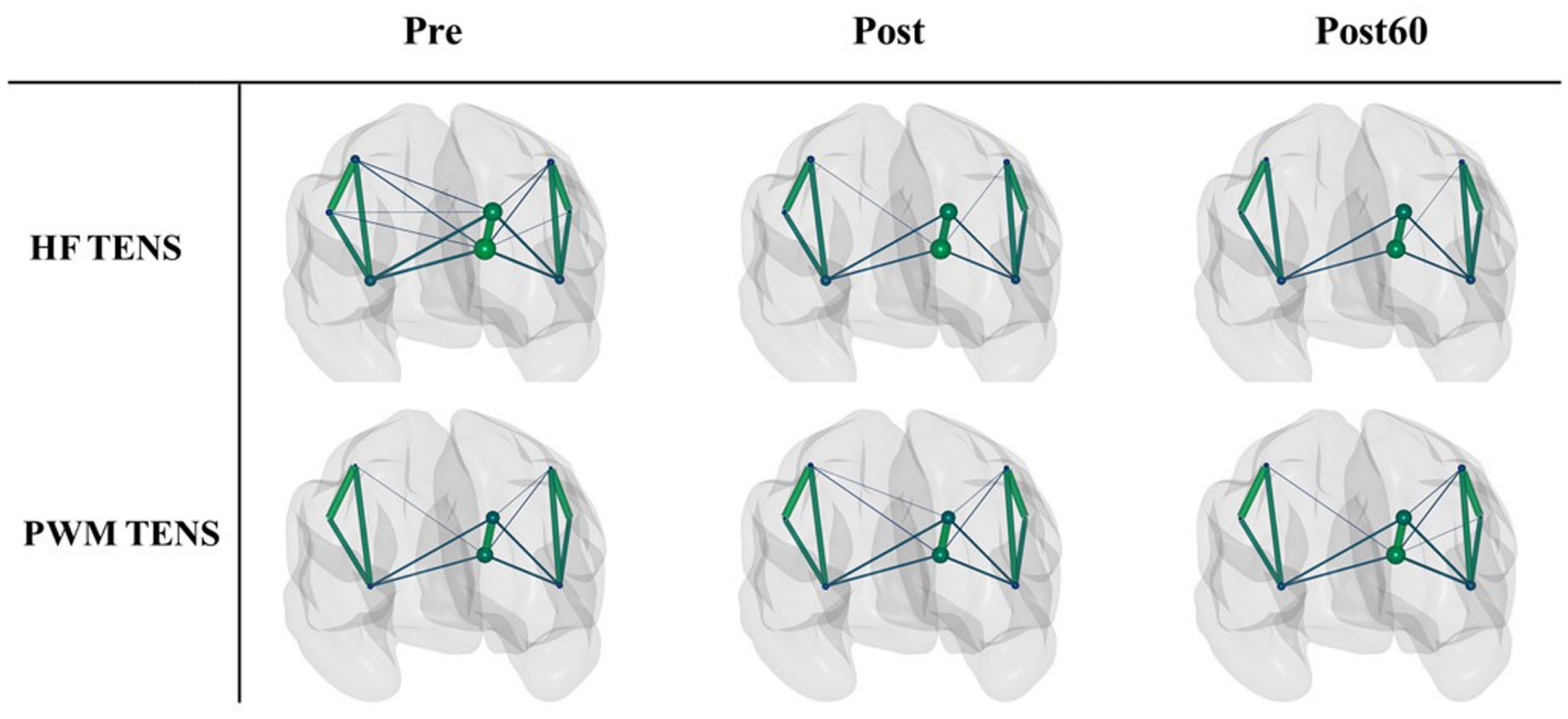
The normalized PLV-based cortical networks in high gamma within three time phases (Pre, Post, and Post60) for HF TENS pattern (first row) and PWM TENS (second row). The size of each node indicates the nodal strength, and the connection between two nodes represents the mean PLV value across subjects normalized to the maximum connection weight. The edges with a value below 0.3 of the overall maximum value were excluded.

The PLV-based cortical FC networks in high gamma-band are shown as an example within the three phases for each TENS pattern in Figure 2. The size of each node indicates the nodal strength, and the connection between two nodes represents the mean PLV value across subjects normalized to the maximum connection weight. To avoid complexity, the edges with a mean value below 30% of the overall maximum value were excluded in the figure.

### 3.1. Local indexes

PLV: The results of the statistical tests on the local characteristics of the PLV-based graph (PLV_G_L_) indicated a significant difference in induced changes by both TENS patterns (i.e., PWM and HF TENS). However, this observation is only statistically valid in the high gamma frequency band one hour following the intervention phase (Post60-Pre). Therefore, the following reported results in this section represent the induced-effect in high gamma frequency band.

**Figure 3:**
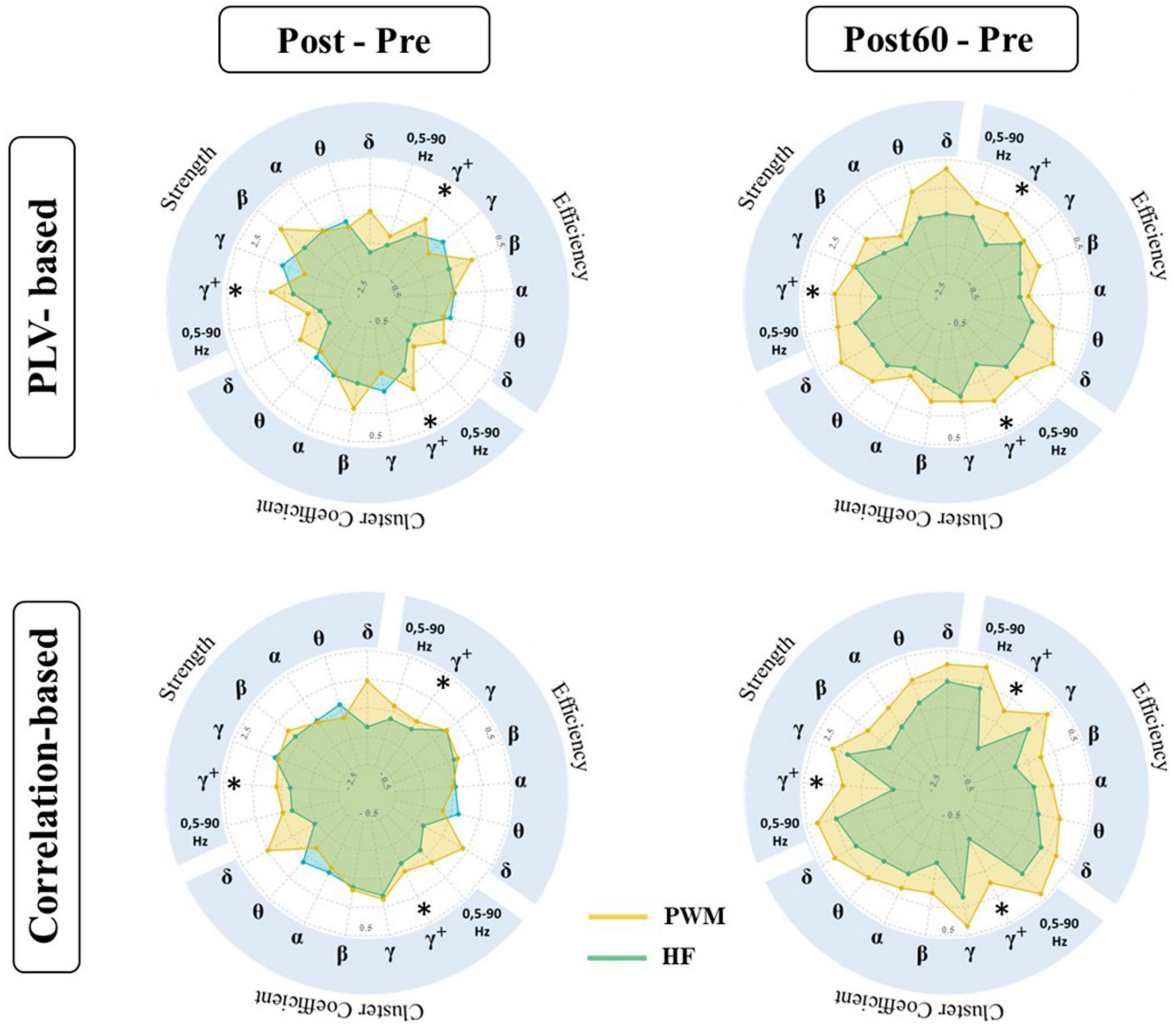
Local characteristics of functional brain network extracted from PLVFC (top row) and CorFC (bottom row) in high gamma-band. Each radar plot represents changes in local indexes by interventions (PWM: orange area and HF: blue area) compared to Pre value (Post – Pre and Post60 – Pre) for each ROIs. Brain regions with significantly different induced changes by two interventions are depicted by *.

As Figure 3 presented, the change in local node strength, efficiency, and cluster coefficient showed a general increasing trend immediately after PWM TENS intervention. In contrast, a slight reduction occurred in PLV_L_G_ indexes right after HF TENS intervention. However, the differences in changes by two patterns were not statistically significant at Post-Pre.

Enhancement in the aforementioned graph measures following PWM TENS was strengthened one hour later and evoked changes by two patterns in nodal strength became significantly different in somatosensory cortices, both contralateral SI_L (*p = 0.013*), SII_L (*p = 0.025*) and ipsilateral SI_R (*p = 0.038*), SII_R (*p = 0.036*) to the TENS side. At the same time, significant differences were detected between alteration of local efficiency by two patterns in contralateral SI_L (*p = 0.030*), SII_L (*p = 0.024*) and ipsilateral SI_R (*p = 0.034*), SII_R (*p = 0.029*), indicating facilitation in information transmission in sensory cortices 60 min after PWM TENS compared with 60 min after HF TENS. Furthermore, enhancement in network cluster coefficient following PWM TENS (at Post) were also observed significantly stronger than induced alteration by HF TENS in mPFC (*p* = 0.048) and ACC (*p = 0.043*) in addition to sensory cortices of both hemispheres, including SI_L (*p = 0*. 026), SII_L (*p = 0.019*), SI_R (*p = 0.029*), and SII_R (*p = 0.026*) sources.

Spearman correlation: Comparative analysis has been also conducted on alteration of the amplitude-based FC network (Cor_L_G_) elicited by TENS interventions (Figure 3). The statistical finding detected significant differences in induced changes at Post60 compared to baseline values (i.e., at Pre) of local node strength, efficiency, and cluster coefficient following PWM and HF TENS in the high gamma-band. It revealed that the effect of two patterns on the local characteristics of the network was not statistically different right after the interventions phase. However, the increase in the local strength of SI_L and SII_L, ACC, and mPFC was observed significantly different with alteration following non-modulated pattern (SI_L; *p = 0.041*, SII_L*; p = 0.032*, ACC*; p = 0.015*, and mPFC; *p = 0.014*). Simultaneously, late enhancement by modulated pattern at Post60 in the local efficiency and cluster coefficient of the network differed statistically with HF TENS-induced supression in SII_L (EF: *p* = 0.023, CC: *p* = 0.028), ACC (*EF: p = 0.019, CC: p = 0.019*), and mPFC (*EF: p = 0.01, CC: p = 0.015*).

### 3.2. Global indexes

**Figure 4:**
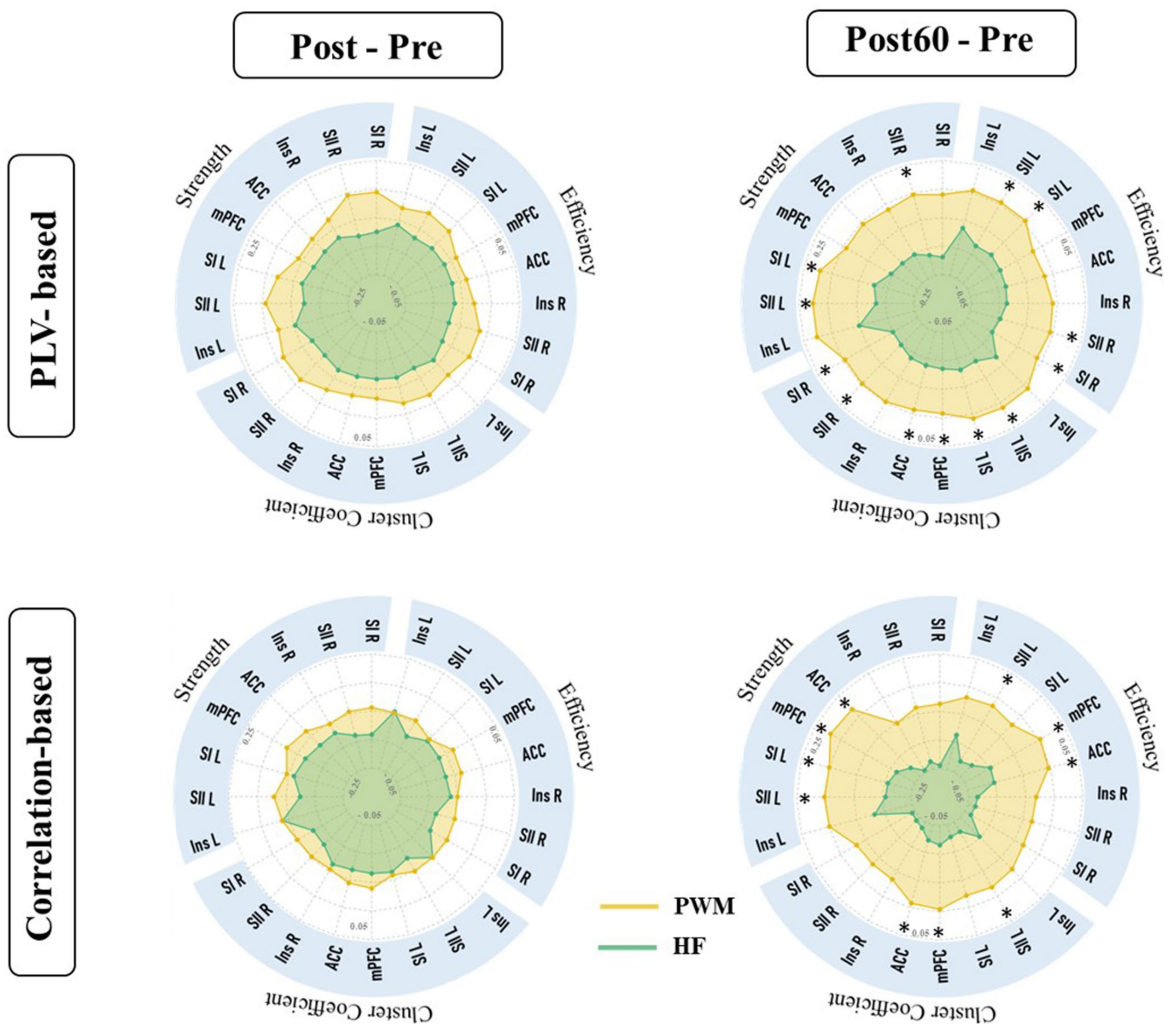
Global characteristics of functional brain network extracted from PLVFC (first row) and CorFC (Second row). Each radar plot illustrates changes in global indexes by interventions (PWM: orange area and HF: blue area) compared to Pre value (Post – Pre and Post60 – Pre) within all frequency bands. Alteration in high gamma global metrics was detected significantly different by two interventions and depicted by *.

In this work, the effect of two TENS patterns was also compared on the global indexes. The alteration of global metrics in each new time phase (i.e., Post-Pre and Post60-Pre) is illustrated in Figure 4. The result was in line with local findings and depicted a significant difference between alterations of global node strength, efficiency, and cluster coefficient elicited by two TENS patterns in only the high gamma-band. It revealed that 60 min after the TENS intervention increase in global metrics of both networks are significantly stronger in the PWM TENS intervention group compared to alteration HF TENS in the high gamma frequency band (Cor_G_G_; S: *p = 0.019*, EF: *p = 0.018*, CC: *p = 0.034* and PLV_G_G_; *S: p = 0.037, EF: p = 0.039, CC: p = 0.031*).

## 4. Discussion

In this study, we aimed to compare the possible alteration in the local and global characteristic of cortical FC network following PWM TENS and HF TENS patterns. The results indicated that PWM TENS alters the cortical FC networks involved in sensory/pain perception, which are statistically different one hour following intervention compared to the changes induced by HF TENS at Post60 only in the high-frequency oscillations (high gamma-band).

Regarding network analysis, local investigation of information exchange quality and quantity between selected ROI and neighbor nodes is widely performed in several neurological conditions, including chronic pain (Farmer et al., 2012; Lenoir et al., 2020; Zheng et al., 2020), and PLP (Ding et al., 2020).

As it is illustrated in Figure 3, induced alteration in local indexes of both amplitude and phase-based cortical network between selected ROIs were not detected statistically significant immediately after the intervention phase. However, the increased local strength and efficiency of the network by PWM TENS in SI and SII sources, contralateral to the TENS side (left hemispheres), were significantly stronger one hour later (Post60 - Pre) compared with HF pattern. The SI cortex is demonstrated to be involved in the sensory-discriminative aspect of pain, including localization and pain intensity, while SII contributes to the recognition of the noxious nature (Bushnell et al., 1999; Timmermann et al., 2001; Xiao et al., 2019). The reduction of interregional FC in sensory cortices has been reported in amputees with PLP. Suppression of intra-hemisphere FC between SI in the affected hemisphere and subcortical nuclei was also shown following amputation (Zhang et al., 2018). Interestingly, a recent clinical study using rTMS with the goal of PLP reduction revealed an increase in FC, mainly in sensory networks and unaffected hemisphere accompanying PLP reduction (Scibilia et al., 2018). Therefore, late enhancement (Post60 - Pre) of local strength and efficiency in left SI and SII, statistically greater than HF TENS-induced changes in our study, might be evidence regarding potentials of PWM TENS with pain and sensation alleviation goals.

Importantly, previous study revealed enhancement in nodal strength of ipsilateral sensory cortices in the TENS group associated with significant sensory suppression compared to the sham group (Zarei et al., 2022). Co-modulation of SI cortices activity between two hemispheres following TENS intervention is considered as a possible explanation. Therefore, findings of this study regarding the increase in local indexes of SI_R and SII_R in the PLV-based network following PWM TENS significantly greater than HF TENS-induced effect may suggest modulated pattern as a possible alternative therapeutic pattern with long-lasting effect for the clinical session applying the TENS intervention to the intact hand, and patients still experienced pain reduction following the intervention phase (Tilak et al., 2016; Peng et al., 2019).

In addition to the sensory cortices, the local cluster coefficient metric also experienced an enhancement in ACC and mPFC regions differing significantly with alteration evoked by non-modulated TENS. Recently Peng et al. showed low-frequency TENS improved functional connectivity between the SI contralateral to the TENS side and mPFC, which was associated with a significant analgesic effect of TENS (Peng et al., 2019). Zarei et al. also revealed the significant contribution of ACC, and mPFC within high gamma oscillations in sensory suppression procedures as a result of conventional TENS intervention (Zarei et al., 2022). Moreover, the chronic pain-evoked reorganization has also been represented in cortical FC beyond the sensory cortices (Zhang et al., 2018). The anterior cingulate cortex (ACC) is known to encode the affective-motivational component of pain, and enhancement of ACC’s aversive responses is associated with chronic pain (Wager et al., 2016; Xiao et al., 2019; Singh et al., 2020). mPFC is also reported as the region of the descending pain inhibitory system, with most neuron projections to the periaqueductal gray (PAG) known as a key midbrain region regulating endogenous pain (Kucyi and Davis, 2015). The increase in the local efficiency of ACC and mPFC regions was recently suggested to play a role in sensation suppression following TENS intervention (Zarei et al., 2022). Therefore, our result illustrated that the significant facilitation in information transmission efficiency of ACC and mPFC occurred 60 min after PWM TENS compared to conventional TENS may support sensation/pain reduction mechanisms by modulated TENS as a therapeutic intervention.

One the other hand, the results revealed significant differences in both phase and amplitude-based global network metrics (PLV_L_G_ and Cor_L_G_) between induced changes 60 min after two TENS patterns in a high gamma-band.

As it is shown in Figure 4, both HF and PWM TENS could not monotonically affect both amplitude and phase-based global nodal strength, efficiency, and clustering coefficient, right after the intervention (Post-Pre) within all frequency bands. However, global network metrics in high gamma-bands increased immediately after the modulated intervention and the enhancement within this band became stronger 60 min later, differing significantly from the alteration induced by HF. In addition, the facilitation trend in the average of global network features was generally observed within all frequency bands one hour after the PWM phase compared with baseline values (Post60 - Pre).

In terms of the global reorganization of brain networks, several studies have shown the major role of this cortical factor in chronic pain (Ta Dinh et al., 2019; Zheng et al., 2020; Lenoir et al., 2021). Recent fMRI research has revealed significant alteration in the global network metrics among patients with chronic pain (Makin et al., 2015). Suppression of high gamma-band global efficiency within the cortical network was indicated to be associated with chronic pain (DeSouza et al., 2020). Moreover, Zheng et al. recently reported the reduced global network efficiency as a pain evoked biomarker in the brain network (Zheng et al., 2020). Tan et al. also showed that the global efficiency of phase-based functional connectivity network (PLV) decreased in high gamma band frequency in chronic pain patients (Ta Dinh et al., 2019). In line with these findings, late enhancement in high gamma global network metrics by PWM significantly stronger than induced alteration by non-modulated TENS in the present study may suggest a new biomarker by modulated TENS mechanism for pain suppression.

Some limitations should also be noted in this study. The authors would like to highlight that the concluded results are based on observation from 12 subjects. Thus, the statistical generalizability of the study could be further improved by conducting a double-blind study design with a larger sample size and by including subjects with diversity in age and gender. This will form one of the future lines of research for this study. Secondly, as we found the effect of PWM TENS for at least an hour following the intervention, in order to enlighten the lasting effect duration for clinical application, later follow-up phases following TENS intervention could be investigated in future studies. Finally, the effect of PWM TENS intervention has been previously studied on corticospinal excitability (Faghani Jadidi et al., 2022) and cortical activity (Jadidi et al., 2023) associated with simultaneous changes on sensory profile and the evidence for the possible underlying mechanism of this pattern on pain modulation has been provided. As a follow-up, here, we investigated the induced alteration by PWM TENS on a higher order level (brain network functional connectivity) in the sensory/pain related brain areas among healthy subjects. Therefore, these findings can be further investigated in a future clinical study in a patient population with chronic pain.

## 5. Conclusion

Recently, utilizing a modulated temporal pattern of TENS (such as PWM TENS) instead of the classical one (specifically HF TENS) has been suggested for pain alleviation. While the effects of PWM TENS on the activity of both sensory and motor cortices highlight this pattern’s potential as an alternative approach for sensory/pain suppression, the underlying mechanism on functional brain connectivity is still unclear. Therefore, in this study, we aimed to conduct a comparative analysis between cortical network alteration following PWM TENS and conventional TENS. The result indicated an increasing trend in global network features lasted one hour following PWM intervention which became statistically significant compared with HF TENS effects in gamma oscillation. Simultaneously, the induced increase in local matrices was detected significantly different between two patterns in several brain regions, namely ACC, mPFC, and SI and SII in both hemispheres. In the present study, discriminative effects by PWM TENS after one hour on the cortical network suggested that while both patterns have potential in pain therapy, late occurred effects by modulated pattern (Post60 - Pre) may highlight it as a future alternative therapeutic intervention in clinical research.

## Data availability statement

The raw data supporting the conclusions of this article will be made available by the authors, without undue reservation.

## Ethics statement

The studies involving human participants were reviewed and approved by The North Denmark Region Committee on Health Research Ethics (N-20190016) in accordance with the Declaration of Helsinki. The patients/participants provided their written informed consent to participate in this study.

## Author contributions

AJ had contribution in design of work, acquisition of data, data analysis, interpretation the result, drafting the work, interpretation, and the finalization of manuscript. WJ contributed to the study design, validation of the methodology, and critical revision of the manuscript. AZ was involved in data acquisition and data analysis. EL has contribution in study design and validation of the methodology. SA has contribution in study design, validation of the methodology, interpretation the result, and critical revision of the manuscript. All authors contributed to the article and approved the submitted version.

## Funding

This project has received funding from the European Union’s Horizon 2020 research and innovation programme under the Marie Skłodowska-Curie grant agreement [754465] and the Center for Neuroplasticity and Pain (CNAP), which is supported by the Danish National Research Foundation [DNRF121].

## Conflict of interest

The authors declare that the research was conducted in the absence of any commercial or financial relationships that could be construed as a potential conflict of interest.

## Publisher’s note

All claims expressed in this article are solely those of the authors and do not necessarily represent those of their affiliated organizations, or those of the publisher, the editors and the reviewers. Any product that may be evaluated in this article, or claim that may be made by its manufacturer, is not guaranteed or endorsed by the publisher.
